# Disease Progression Mediated by Egr-1 Associated Signaling in Response to Oxidative Stress

**DOI:** 10.3390/ijms131013104

**Published:** 2012-10-12

**Authors:** Judith-Irina Pagel, Elisabeth Deindl

**Affiliations:** Walter-Brendel-Centre of Experimental Medicine, Ludwig-Maximilians-University, Munich D-81377, Germany; E-Mail: elisabeth.deindl@med.uni-muenchen.de

**Keywords:** Egr-1, oxidative stress, atherosclerosis, NADPH oxidase inhibitor, pulmonary hypertension, signal transduction, MEK/ERK pathway, PKC

## Abstract

When cellular reducing enzymes fail to shield the cell from increased amounts of reactive oxygen species (ROS), oxidative stress arises. The redox state is misbalanced, DNA and proteins are damaged and cellular transcription networks are activated. This condition can lead to the initiation and/or to the progression of atherosclerosis, tumors or pulmonary hypertension; diseases that are decisively furthered by the presence of oxidizing agents. Redox sensitive genes, like the zinc finger transcription factor early growth response 1 (Egr-1), play a pivotal role in the pathophysiology of these diseases. Apart from inducing apoptosis, signaling partners like the MEK/ERK pathway or the protein kinase C (PKC) can activate salvage programs such as cell proliferation that do not ameliorate, but rather worsen their outcome. Here, we review the currently available data on Egr-1 related signal transduction cascades in response to oxidative stress in the progression of epidemiologically significant diseases. Knowing the molecular pathways behind the pathology will greatly enhance our ability to identify possible targets for the development of new therapeutic strategies.

## 1. Introduction

Redox reactions contribute to countless significant biological processes and help to maintain vital cellular functions. During aerobic cellular respiration, for instance, glucose is oxidized to CO_2_ and oxygen is reduced to water. This key process is elementary to gain energy in the form of adenosine triphosphate (ATP). Coenzymes like nicotinamide adenine dinucleotide (NAD^+^) participate in these electron transfer reactions and can act as oxidizing (NAD^+^) or reducing agents (NADH). NADH can be further metabolized during oxidative phosphorylation, the electron transport chain across the inner mitochondrial membrane, to generate more ATP. Its related cofactor NAD phosphate (NADP^+^) is reduced to NADPH during the oxidative phase of the pentose phosphate pathway (PPP) in the cytosol. NADPH is an essential reducing agent, not only for anabolic reactions like nucleic acid synthesis, but also for the production and elimination of reactive oxygen species (ROS). ROS—as the name implies—are a heterogenic group of highly reactive molecules and a normal byproduct of metabolic redox reactions [1]. Among them are free radicals, which hold an unpaired valence shell electron, like superoxide (^•^O_2_^−^), nitric oxide (NO^•^) or the hydroxyl radical (^•^OH), and oxidizing molecules such as hydrogen peroxide (H_2_O_2_) or hypochlorus acid (HOCl). ROS are important mediators of cell signaling (called redox signaling) [2] and are indispensable for the immune defense in macrophages. The enzyme NADPH oxidase, for example, generates superoxide from molecular oxygen, which is then used to kill bacteria in the respiratory burst reaction within the cell [3]. Nonetheless, high levels of ROS contribute to cell toxicity [4], since they also damage host DNA/RNA [5], oxidize proteins [6] or cause lipid peroxidation [7]. Therefore, the proper balance (redox state) between ROS production and consumption must be maintained within the cellular compartments. Enzymes such as superoxide dismutase (SOD), catalases or peroxidases are capable of metabolizing accumulated ROS. Catalase, for example, decomposes H_2_O_2_ to H_2_O and O_2_. Glutathione uses NADPH to reduce H_2_O_2_ amounts and to regenerate itself. Other antioxidants, such as ascorbic acid (vitamin C) and tocopherol (vitamin E), are described as important radical scavengers [8]. The imbalance between the production of metabolically derived ROS and the organism’s deficiency to detoxify the cell and to repair the acquired cellular damage is called oxidative stress. This condition is related to diseases like atherosclerosis, diabetes, pulmonary hypertension (PH), cancer or Alzheimer’s disease [9–11]. Constantly increased amounts of oxidizing agents activate various signaling pathways that in turn are targeting the promoters of “redox sensitive” genes. One of these genes encodes the zinc finger transcription factor early growth response 1 (Egr-1). ROS have been shown to rapidly induce Egr-1 mRNA and protein expression [12]. Available data focusing on Egr-1 signaling after oxidative stress is limited and most of it is based on *in vitro* experiments [12–15]. Egr-1’s involvement in these epidemiologically relevant diseases, however, is important to understand and to develop new therapeutic strategies. Here we review the current data on Egr-1 in response to oxidative stress in the context of pathology.

## 2. Egr-1—A Redox Sensitive Transcription Factor

### 2.1. Structural Properties of Egr-1 Protein

Two exons code for an 80–82 kDa Cys_2_-His_2_-type zinc-finger transcription factor mapping to chromosome 5 [16]. Egr-1 was found to be rapidly and transiently expressed in response of a heterogenic group of stimuli like growth factors (GFs) [17], shear stress [18], oxygen deprivation [19,20], (reperfusion) injury [21–23] and oxidative stress [12,24,25]. The central DNA binding domain (DBD) of Egr-1 consists of the three zinc-finger motives [26,27] that characteristically bind to GC-rich promoter sequences (GCG(G/T)GGCG), therefore named Egr binding sequence (EBS) [17]. Egr-1 interconnects a broad variety of cascades upstream and downstream.

### 2.2. Functional Motifs at the Promoter

Besides an EBS [28], several functional response elements on the Egr-1 promoter presenting targets of distinct signal transduction cascades have been investigated and characterized [29]. At the 3′ end of the promoter and next to the TATA box, five serum response elements (SRE) are located [30]. Five Ets-family transcription factor-binding sites are arranged adjacently to these SREs. Furthermore, two cyclic adenosine monophosphate (cAMP) response elements (CREs), an APETALA1 (AP1) and two gene-specific activator protein 1 (Sp1) binding sites have been described [31].

### 2.3. Redox Regulated Transcription Capability

Previous *in vitro* studies described that redox levels influence the DNA binding capacity of Egr-1 in a dose-dependent manner. Cys residues within the DNA-binding domain of the protein are oxidized and severely diminish the DNA binding capacity of Egr-1, whereas under reducing conditions, DNA binding is enhanced [15]. Under non-toxic ROS levels, Egr-1’s binding ability remains preserved by activation of an apurinic/apyrimidinic endonuclease 1 (APE1) [15,25]. APE1 is a DNA repair enzyme with nuclear redox activity [32–34]. In various cell types, ROS induce nuclear translocation of APE1 [35,36], which in turn induces DNA binding of transcriptional regulators. APE1 restores Egr-1 DNA binding by direct protein—to protein interactions without neosynthesis and subsequently enhances its transcriptional activity; most likely via posttranslational modification [25]. Evidence for a positive autoregulatory loop between APE1 and Egr-1 exists [25]. Egr-1 upregulates APE1 by protein neosynthesis and APE1 in turn preserves the DNA-binding capacity of Egr-1, therefore mutually maintaining their transcriptional activity under non-toxic redox conditions. However, to prevent a never-ending activation between APE1 [37,38] and Egr-1 [28,39], the autoregulatory loop will eventually shut down, since APE1 binding to its own promoter leads to a downregulation by its own product [37].

### 2.4. MAPK Signal Transduction Cascades Aiming at the SRE

Hydrogen peroxide at non-toxic doses was shown to upregulate Egr-1 mRNA *in vitro* [12]. Moreover, Egr-1 activation was demonstrated to be MEK/ERK and c-Jun *N*-terminal kinases (JNKs) dependent in H9c2 cells; a myogenic cell line derived from the embryonic rat ventricle [40]. P42/44 mitogen-activated protein (MAP) kinase (MAPK), also known as extracellular signal-regulated kinase (ERK) 1/2, is part of the classical MAPK pathway being described to activate the Egr-1 promoter [41,42]. ERK1/2 has been shown to be phosphorylated and therefore directly activated through high levels of ROS [43], such as xanthine oxidase derived H_2_O_2_ [41], though the underlying mechanism still remains to be elucidated. Raf, mitogen-activated protein kinase kinase kinase (MAPKKK), activates mitogen-activated protein kinase kinase (MAPKK = MEK) 1/2, which in turn phosphorylates the dual acceptor motif ERK1/2 (Thr-Glu-Tyr). Activated ERK translocates into the nucleus and promotes binding of Ets-family transcription factor Elk-1 to the DNA. In close proximity to Elk-1, the SREs binding sites are located, indicating mutual activation. Serum response factor (SRF) is a MADS (**M**cm1 and **A**rg80 in yeast, **A**gamous and **D**eficiens in plants, **S**RF in animals) -box transcription factor targeting SRE binding sites, which contain the characteristic sequence CC(A/T)_6_GG also known as CArG-box [44]. SRF has been shown to be involved in the transcriptional regulation of various GF-inducible genes [45,46], among them Egr-1 [47]. SRF is classically dependent on binding of the ternary complex factors (TCFs) Elk-1, Sap1 and Sap2, to activate transcription [45]. Elk-1 and SRF form a ternary complex [48] and together activate transcription [49,50] combining the MEK/ERK pathway with SRF associated gene regulation. There are two other MAPKs, associated with mechanical stress, that have been found to interact with Egr-1 [51]. These are JNKs, referred to as stress-activated protein kinases (SAPKs) and p38 isoforms. Both of them are responsive to mechanical, oxidative or environmental stress [52]. The p38 kinase, however, was not found to be involved in H_2_O_2_ dependent upregulation of Egr-1 [42].

## 3. Egr-1 Mediated Proliferation in Hypoxia Induced Pulmonary Fibrosis and Hypertension

Egr-1 is highly associated with growth, vascular cell proliferation [53], cell survival programs [18,54] and apoptosis [55]. A proliferative response to H_2_O_2_ is thought to be a protective mechanism against oxidant injury. Signal transduction of the H_2_O_2_-induced mitogenic signaling has been described to occur *via* the activation of MAPK and to increase the expression of Egr-1 in aortic smooth muscle cells [41]. Egr-1 regulates the expression of transforming growth factor beta 1 (TGF-β_1_) [56] and *vice versa* [57]. In an *in vivo* model of pulmonary fibrosis, TGF-β_1_ promotes epithelial apoptosis followed by mononuclear-rich inflammation, tissue fibrosis, myofibroblast and myocyte hyperplasia [55]. A null mutation of Egr-1 blocked TGF-β_1_ induced apoptosis *in vivo*, ameliorating collagen content, alveolar remodeling and parenchymal leukocyte infiltration [55]. Production of ROS has been implicated in chronic hypoxia-induced pulmonary hypertension (PH) and pulmonary vascular remodeling. Superoxide, generated under hypoxic conditions, contributed to PH through the induction of Egr-1 and its downstream gene target, tissue factor (TF) [58]. Egr-1 has been described to further the hypoxia induced autonomous proliferation of pulmonary artery adventitial fibroblasts via upregulation of the cell cycle regulator cyclin D, a key mechanism in the progression of disease [59]. Chronic hypoxia decreased lung SOD activity and SOD overexpression attenuated chronic hypoxic PH and vascular remodeling. Endothelial cell (EC) derived SOD (EC-SOD) overexpression also prevented the early hypoxia-dependent upregulation of Egr-1 and the procoagulant protein TF [58].

## 4. Apoptosis and Tumorigenesis

Apoptosis induced by H_2_O_2_ is thought to be a direct consequence of oxidant injury. Cellular Abelson murine leukemia viral oncogene homolog (c-Abl) is a tyrosine kinase that can act as a regulator of cell growth and apoptosis in response to oxidative stress. Significantly, H_2_O_2_-induced Egr-1 expression *in vitro* seems also to be induced by c-Abl kinase activity. Furthermore, c-Abl aims at the three distal SREs on the Egr-1 promoter via the MEK/ERK signaling. In addition, c-Abl-induced apoptosis is partially mitigated by Egr-1 activity, as cells, devoid of Egr-1 expression, undergo reduced rates of c-Abl-induced apoptosis [60].

When a transcription factor is participating in cell cycle control as a physiologic response to hypoxia or injury [61,62], an association with tumor growth is likely to be suspected. A number of tumor supressor genes are regulated directly by Egr-1, among them p53 [56], and the already mentioned relation betweeen GFs and Egr-1 has also been described for tumor dependent angiogenesis [63]. Cells expressing the breakpoint cluster region-abelson (bcr-Abl) oncogene demonstrate increased levels of intracellular ROS [64] and signaling initiated by the bcr-Abl kinase causes chronic myelogenous leukemia (CML). A recent publication reported that transcriptional upregulation of Fyn, a ROS sensitive src-family member, was strongly dependent on Egr-1 in an *in vitro* model [65], indicating participation of Egr-1 in the pathogenesis of CML.

In a majority of human prostate carcinoma specimens Egr-1 protein expression control was lost, suggesting that high levels of Egr-1 plays a central role in the initiation of human prostate cancers [66]. Indeed it was evidenced that Egr-1 deficient mice demonstrated impaired prostate tumor growth [67]. Alterations in the androgen receptor signaling were found to be a major cause of the disease and it has been shown that Egr-1 promotes the translocation of the androgen receptor into the nucleus [68]. Anti-hormonal therapy of prostate cancer becomes limited in the state of androgen-independent disease [69] and Egr-1 seems also capable to govern prostate cancer progression under androgen resistance [70,71]. Therefore, Egr-1 might be a new and interesting target of anti tumor therapy especially when anti-hormonal drugs are no longer effective.

## 5. Involvement of Egr-1 in Viral Pathogenesis

Several viral infections lead to the activation of Egr-1 [72–74]. Infections with the *Herpesviridae* family are not only characterized by a high prevalence in the human population, such as herpes simplex virus 1 and 2 (HHV1/2) [75], but also have specifically been described to promote tumors such as the Eppstein- Barr Virus (HHV4) or the Kaposi’s sarcoma-associated herpesvirus (KSHV) also known as HHV8. High stress levels can trigger and reactivate viral infections that have been latent for a long time or even can promote virus-associated malignancies on the long term [76,77]. Egr-1 has also been shown to critically participate in the KSHV reactivation process directly by mediating transcription of the gene encoding for replication and transcription activator (RTA) [78], a viral component known to control the switch from latent to lytic infection [79,80]. This seems also to be true for the reactivation of EBV, where Egr-1 has also been shown to positively regulate RTA via a positive feedback mechanism [81].

## 6. Signaling Involved in the Pathogenesis of Atherosclerosis

### 6.1. NADPH-Oxidase, Hemin and Egr-1

As mentioned above, NADPH-oxidase is a ROS generating enzyme. Many different stimuli including angiotensin II, glucose, and oxidized LDL activate NADPH-oxidases in the vascular wall [82]. Furthermore, NADPH-oxidase was found in coronary specimens and a relationship between plaque formation and NADPH-oxidase expression could be detected [83]. Extracorpuscular heme (ferroprotoporphyrin IX) released from hemoglobin is a potent proinflammatory ROS inducer. Heme catalyzes the oxidation of LDL, thus promoting foam cell formation and vascular smooth muscle cell (vSMC) proliferation. Hemin, oxidized heme, has been found to mediate redox-sensitive gene expression and to contribute to atherosclerotic plaque progression. Via MEK/ERK, hemin upregulated Egr-1 in vSMCs. This was directly dependent on NADPH-oxidase activity. The novel NADPH-oxidase inhibitors apocynin and diphenyleneiodonium chloride were also tested and could block hemin induced Egr-1 expression [84].

### 6.2. PKC and Egr-1

The role of Egr-1 for the pathogenesis of atherosclerosis is quite well described [85]. The protein kinase C (PKC) isoforms have been shown to lie upstream of Egr-1 [86]. In human aortic smooth muscle cells, PKCβ_II_ and PKCΦ activation and MEK/ERK mediated Egr-1 expression were essential for low dense lipoprotein (LDL)-induced cell proliferation [87], furthering the progression of the disease. It is well established, that H_2_O_2_ also leads to PKC activation [88] and *in vitro*, the autoregulatory loop between APE1 and Egr-1 was shown to be PKC dependent [25]. In an *in vivo* study, atherosclerosis was markedly impaired in mice deficient for both PKCβ and ApoE when compared to ApoE null mice [89]. Finally, recent reports have shown that gastrin effects on Egr-1 expression were dependent on activation of PKC family kinases, but do not require Ras (as involved in GF mediated MEK/ERK activation), phosphoinositol-3-kinase (PI3K) or intracellular calcium signals and are therefore arguing for a PKC/Raf/MEK/ERK/Egr-1 pathway [90].

### 6.3. Egr-1 and Accelerated Atherosclerosis in Diabetic Disease

It has been shown that insulin stimulates Egr-1 protein expression in endothelial cells (ECs) [91] and vSMCs [92] via the MEK/ERK pathway. Moreover, oxidative stress combined with insulin as initial stimulus further enhanced Egr-1 activation. Insulin resistance is characterized by compensatory hyperinsulinemia, with a functional MEK/ERK signaling but with selective impairment of PI3K [93]. Glucose can also induce Egr-1 expression, but PKC dependent in EC [91]. Egr-1 influences on the insulin gene itself could be mediated *via* pancreas duodenum homeobox-1 (PDX-1) [94,95], important for glucose homeostasis. Egr-1 signaling, PI3K or PKC might be interesting targets for drug therapy in atherosclerosis and diabetic disease in the future.

## 7. Conclusion—Daily Antioxidants as Dietary Supplements for the Prevention of Disease?

**Figure 1:**
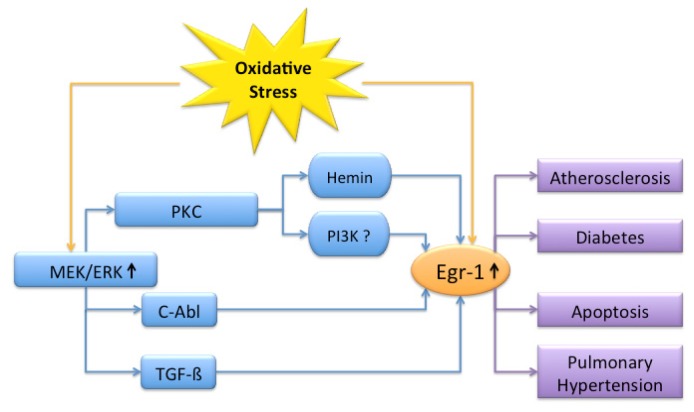
Overview of signaling partners involved in oxidative stress mediated Egr-1 signaling. Oxidative stress leads to Egr-1 activation (↑) and promotes atherosclerosis, diabetes, apoptosis and pulmonary hypertension. The MEK/ERK pathway is the main signal transduction cascade involved. Depending on the targeted cell type and *in vitro* or *in vivo* data, different elements are involved.

Oxidizing agents such as ROS or free radicals can be initiators and mediators of disease. When natural detoxifying enzymes fail, oxidative stress occurs and may cause aging, atherosclerosis or tumors. Egr-1 is rapidly induced after exposure to oxidants and interacts with various signaling partners mostly in the direction of disease progression (Figure 1).

Since Harman postulated the free radical theory of aging (FRTA) in 1956 [96], extensive research has been conducted to discover the key to a longer lifespan. Prevention of the formation of oxide radicals became an important target of the pharmaceutical industry and today a large group of over-the-counter drugs flood the drug market. But can the largely propagated daily supply of antioxidants, like vitamin C, E or selenium, in form of pills in fact reduce the incidence or outcome of a disease? In recent meta-analyses, the authors analyzed clinical trials investigating the oral supply of vitamins in tablet form on quality of life, mortality or the incidence of cardiovascular diseases and colon cancer. Unfortunately, they came to the conclusion that these high doses of antioxidants had no proven positive effect or even led to an increased mortality [97–100]. By scavenging ROS, fine-tuned feed back mechanisms such as the APE1/Egr-1 relation may become deranged. In turn, reducing ROS levels might also increase Egr-1 binding activity and even promote tumor progression or atherosclerosis. Therefore, the approach of disease prevention by scavenging radicals with high-dose supplements is neither reasonable nor safe. Probably we underestimate the complexity of fine regulated cellular signals in oxygen metabolism and in diseases. NADPH-oxidase inhibitors are currently in the very early stages of development and serious side effects regarding immune competence might be expected. The best guideline for a longer lifespan and the prevention of disease is a healthy lifestyle. This involves a balanced diet with high intake of fiber and natural antioxidants found in fruit and vegetables as well as daily physical activity and the cessation of smoking. Further studies at the molecular level are necessary to dissect the pathophysiological mechanisms behind ROS-induced signaling. Not until then, will we be able to design a distinct and individually matched therapy that will help to improve the outcome of diseases induced by oxidative stress.
